# Thromboembolic risk of electrical cardioversion in patients with cardiogenic shock

**DOI:** 10.1016/j.ijcha.2025.101835

**Published:** 2025-11-06

**Authors:** Jonas Gmeiner, Lea Haag, Enzo Lüsebrink, Jan-Philipp Simon, Felix Michelson, Elina Oesterhaus, Wolf-Stephan Rudi, Ludwig Weckbach, Stefan Kääb, Michael Näbauer, Sven Peterß, Christopher Stremmel, Steffen Massberg, Martin Orban, Moritz F. Sinner, Clemens Scherer

**Affiliations:** a Department of Medicine I, University Hospital, LMU Munich, Munich, Germany; b Department of Medicine II, University Hospital Bonn, Bonn, Germany; c Department of Medicine I, University Hospital, LMU Munich, Munich, Germany & German Centre for Cardiovascular Research (DZHK), partner site: Munich Heart Alliance, Munich, Germany; d Department of Cardiac Surgery, University Hospital, LMU Munich, Munich, Germany

**Keywords:** Atrial fibrillation, Atrial flutter, Cardiogenic shock, Cardioversion, Stroke

## Abstract

**Background:**

Cardiogenic shock patients with atrial fibrillation or flutter often require acute cardioversion despite absence of sufficient anticoagulation or the possibility to rule out left atrial appendage thrombus. Thromboembolic risk in these patients is unknown.

**Methods:**

In this study, all cardiogenic shock patients from the LMUshock registry undergoing electrical cardioversion for atrial fibrillation or atrial flutter were included. The primary endpoint was new thromboembolic stroke or systemic embolism at 30 days. Secondary endpoints included performance of transesophageal echocardiography, all-cause mortality and bleeding according to BARC.

**Results:**

Of 140 patients undergoing electrical cardioversion, 36 had preexisting and 104 experienced new onset of atrial fibrillation or flutter during ICU stay. Of these, 87.1 % had anticoagulation with unfractionated heparin and anticoagulation was adjudicated sufficient in 44.3 % at the time of cardioversion. Transesophageal echocardiography was performed in 37.9 % of patients before cardioversion. The primary endpoint was met in 3 patients (2.1 %), all of which had insufficient anticoagulation. All-cause mortality at 30 days was 37.9 % and bleeding ≥ BARC type 3a was found in 12.9 %.

**Conclusions:**

Thromboembolic risk of electrical cardioversion was low despite the limited utilization of transesophageal echocardiography. This may be attributed to the routine administration of therapeutic anticoagulation in this study, but a high incidence of bleeding was observed.

## Highlights

• In cardiogenic shock, stroke risk after cardioversion was low.
• Thromboembolic events occurred only with insufficient anticoagulation.
• Transesophageal echocardiography was rarely performed before cardioversion.
• Severe bleeding occurred in about one in eight patients.

## Graphical abstract

Visual summary of the study showing patient cohort, endpoints as well as results.

**Figure:**
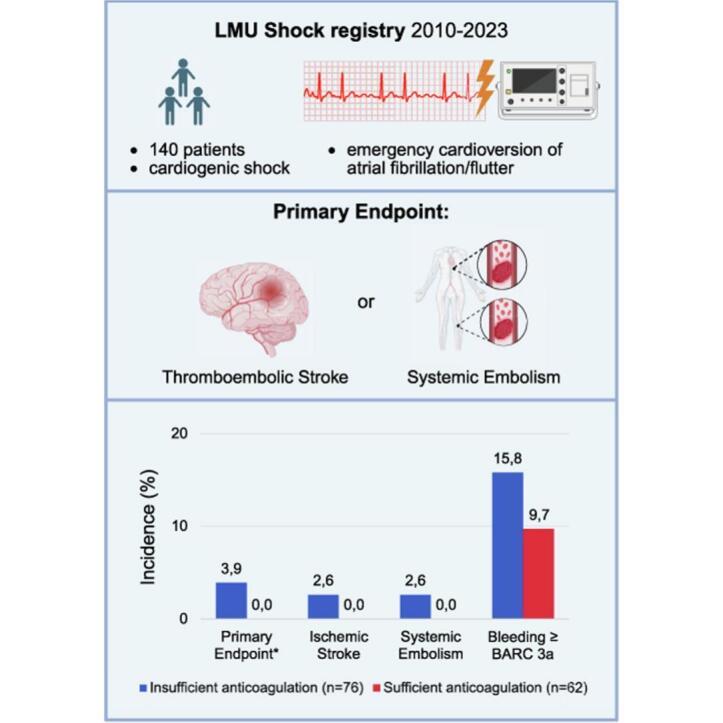

## 1 Background

A major complication of electrical cardioversion for atrial fibrillation (AF) or atrial flutter is ischemic stroke. Guidelines recommend three weeks of uninterrupted, adequately dosed oral anticoagulation or transesophageal echocardiography (TEE) to reduce the risk of left atrial thrombi as a source of thromboembolic events prior to cardioversion.^(1)^ Only in cases of short duration of AF, i.e. < 48 h, early cardioversion without TEE guiding or oral anticoagulation may be performed. Yet, an increased risk of stroke for an AF duration of > 12 h was suggested [1,2].

Cardiogenic shock (CS) patients not only have a high prevalence of AF at hospital admission, but also frequently develop AF while hospitalized [3,4]. Moreover, they are at increased risk of thromboembolic events for several reasons: CS patients usually present with a high comorbidity burden that elevates the risk of thromboembolic events. Heart failure confers impaired hemodynamics resulting in an acutely low blood flow in the left atrium. A hyperinflammatory and hypercoagulatory state can be observed in acute heart failure and might further aggravate the thromboembolic risk [5].

In CS patients, rate control strategies of AF are limited by the negative inotropic effect of betablocker or calcium channel blocker therapy. Hence, CS patients often require urgent cardioversion to stabilize hemodynamics without the possibility of performing TEE or of maintaining prolonged intervals of sufficient anticoagulation. However, data on the thromboembolic risk of cardioversion in this patient group is lacking, leaving ICU personnel without evidence-based guidance. As a consequence, urgently required cardioversion might be postponed. Therefore, the aim of our study was to investigate the thromboembolic risk of cardioversion for AF or atrial flutter in patients presenting with CS.

## 2 Methods

### 2.1 Study population

All patients treated for CS in the cardiac intensive care unit (ICU) of the LMU University Hospital between January 2010 and December 2023 are included in the LMUshock registry (WHO International Clinical Trials Registry Platform Number DRKS00015860). For the present analysis, we retrospectively selected all patients undergoing electrical cardioversion for AF or atrial flutter during the ICU stay. Additionally, the number of patients that had a diagnosis of LAA thrombus during the ICU stay was assessed to estimate the number of cardioversions aborted after TEE revealed LAA thrombus. Data collection and analysis was in accordance with the Declaration of Helsinki and German data protection laws. The study was approved by the local ethics committee (IRB number: 18–001). CS was defined according to the ESC clinical practice guidelines, the IABP-SHOCK II trial and the CULPRIT SHOCK trial: [[6], [7], [8]].

- Hypotension despite adequate filling status (systolic blood pressure < 90 mmHg for ≥ 30 min or catecholamine support to maintain systolic blood pressure > 90 mmHg or need for mechanical circulatory support)
- signs of pulmonary congestion
- one of the following clinical and laboratory signs of hypoperfusion:
  o Clinical: altered mental status, dizziness, cold, clammy skin and extremities, oliguria with urine output less than 30  mL/hr, narrow pulse pressure
  o Laboratory: metabolic acidosis, elevated serum lactate greater than 2 mmol/L, elevated creatinine, due to primary cardiac dysfunction

### 2.2 Electrical cardioversion

All enrolled patients received at least one electrical cardioversion. Additional shocks administered within 15 min were considered as one single cardioversion. In case of multiple cardioversion sessions for the same patient during the ICU stay, only the first session was included for analysis. As per standard of our cardiac ICU care, exclusion of left atrial appendage (LAA) thrombi by TEE was performed prior to cardioversion unless precluded by hemodynamic instability, unless the duration of AF or flutter was ≤ 1 day, or unless uninterrupted, sufficient anticoagulation was documented since the arrhythmia onset.

### 2.3 Study endpoints

The primary endpoint was the incidence of new thromboembolic events at 30 days. Thromboembolic events included ischemic stroke or systemic embolism. Secondary endpoints included ischemic stroke, systemic embolism, all-cause mortality at 30 days, bleeding and performance of TEE. Bleeding complications after cardioversion were graded according to the Bleeding Academic Research Consortium (BARC) [9]. Bleeding ≥ type 3a (hemoglobin drop ≥ 3 g/dl or need for transfusion) was considered clinically significant. In case of discharge earlier than a minimum of 30 days, a telephone interview was scheduled to obtain endpoint information.

### 2.4 Anticoagulation

Unfractionated heparin for a target partial thromboplastin time of 60–80 s was the standard therapeutic anticoagulation regime in our cardiac ICU. Alternative anticoagulation strategies were acceptable at the discretion of the treating physician.

For this analysis, anticoagulation with unfractionated heparin was considered sufficient if partial thromboplastin time was increased to ≥ 60 s or a bolus of ≥ 2000 IU of unfractionated heparin was administered shortly before cardioversion. Anticoagulation with vitamin-k antagonists was deemed sufficient if INR was ≥ 2.0. Anticoagulation with low molecular weight heparin and direct oral anticoagulation was considered sufficient if recommended doses for therapeutic anticoagulation were administered.

### 2.5 Statistical analysis

Statistical analysis was performed in accordance with the STROBE (Strengthening the Reporting of Observational Studies in Epidemiology) statement using SPSS (version 28.0.0.0, Chicago, IL, USA) [10]. Continuous variables were reported as mean ± standard deviation or as median with interquartile range as appropriate. Categorical variables were reported as absolute numbers and percentages. In case of missing data for a given variable, the number of cases with missing data was stated in the respective results section.

## 3 Results

**Fig. 1:**
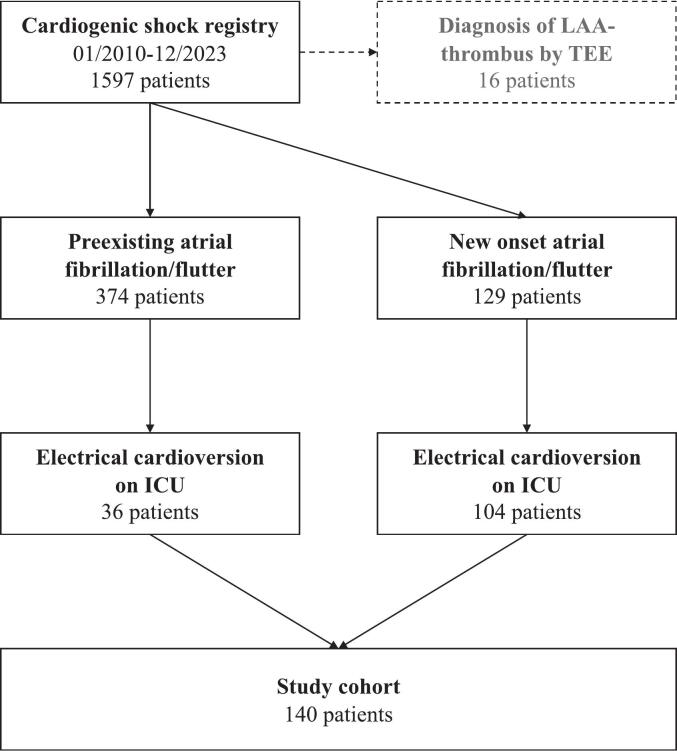
**Study flow chart.** Flow chart showing the number of patients included in the study stratified by onset of atrial arrhythmia.

The study flow chart is visualized in Fig. 1. Of 1597 CS patients in the registry, 503 patients had AF or flutter. Of these, 140 patients underwent electric cardioversion and were included in the study. Further, TEE studies performed on the ICU lead to a diagnosis of 16 LAA thrombi.

Baseline characteristics of the cohort are shown in Table 1. Patients were presenting with a high thromboembolic risk according to CHA_2_DS_2_-VASc-Score (median score 4 (2–5) points) and 41.4 % had CS complicating myocardial infarction. On admission, patients were severely ill. Mechanical ventilation was required in 49.3 % and approximately one quarter required device-based mechanical circulatory support. The mean arterial lactate on admission was 4.1 (2.2–9.9) mmol/L.

**Table 1:** Patient characteristics.

	n = 140
**Past medical history**	
Age	66.0 (56.3–74.0)
Male sex	109 (77.9)
Smoker	
Active smoker	42 (30.0)
Former smoker	24 (17.1)
Never smoker	74 (52.9)
Hypertension	91 (65.0)
Dyslipidemia	61 (43.6)
Diabetes mellitus	46 (32.9)
Previous myocardial infarction	36 (25.7)
Previous percutaneous coronary intervention	44 (31.4)
Previous coronary artery bypass graft	12 (8.6)
Previous stroke	13 (9.3)
Peripheral arterial disease	15 (10.7)
Previous atrial fibrillation/flutter	36 (25.7)
CHA_2_DS_2_-VASc-Score	4 (2–5)
1	11 (7.9)
2	33 (23.6)
3	22 (15.7)
4	36 (25.7)
5	19 (13.6)
6	13 (9.3)
7	2 (1.4)
8	2 (1.4)
9	2 (1.4)
**Status on admission**	
Etiology of cardiogenic shock	
STEMI	28 (20.0)
NSTEMI	30 (21.4)
Non acute coronary syndrome	82 (58.6)
Out of hospital cardiac arrest	18 (12.9)
Mechanical ventilation	68 (49.3)
Veno-arterial extracorporeal membrane oxygenation (VA-ECMO)	31 (22.1)
Percutaneous transvalvular microaxial flow pump (Impella®)	8 (5.7)
Ejection fraction (%)	27.0 (20.0–37.0)
eGFR (ml/min)	38.0 (24.0–60.0)
Lactate (mmol/L)	4.1 (2.2–9.9)

Values are depicted as absolute numbers (percentage of total numbers), mean +- standard deviation or median (interquartile range) as appropriate.

ACS = acute coronary syndrome.

eGFR = estimated glomerular filtration rate.

NSTEMI = non-ST-elevation myocardial infarction

STEMI = ST-elevation myocardial infarction

VA-ECMO = venoarterial extracorporeal membrane oxygenation

Details on cardioversion procedures and anticoagulation treatment are shown in Table 2 and Table 3, respectively. Most patients underwent cardioversion for AF (79.3 %). TEE prior to cardioversion was performed in 37.9 % of patients. Unfractionated heparin was predominantly used for anticoagulation (87.1 %) and 44.3 % of patients received anticoagulation that was adjudicated sufficient at the time of cardioversion. Four patients were discharged alive and lost to follow up thereafter, accounting for a complete 30-days follow up in 97 % of patients.

**Table 2:** Electrical cardioversion details.

Indication for cardioversion	
Atrial fibrillation	111 (79.3)
Atrial flutter	29 (20.7)
Number of shocks	
1	92 (65.7)
2	27 (19.3)
≥3	21 (15.0)
Successful cardioversion to sinus rhythm	98 (70.0)
Recurrence of atrial arrhythmia during hospital stay^a^	44 (44.9)
TEE prior to cardioversion	53 (37.9)
Amiodaron	
oral medication	40 (28.6)
i.v. bolus during cardioversion^b^	40 (28.5)
Betablocker i.v. during cardioversion^b^	6 (4.3)

Values are depicted as absolute numbers (percentage of total numbers).

i.v. intravenous.

eCV = electrical cardioversion

i.v. = intravenous.

a refers to patients with successful cardioversion only.

b up to 5 h before cardioversion

**Table 3:** Details of anticoagulation treatment.

Anticoagulation	
Unfractioned heparin	122 (87.1)
Low molecular weight heparin	2 (1.4)
Direct oral anticoagulation	5 (3.6)
Phenprocoumon	2 (1.4)
Argatroban	1 (0.7)
None	5 (3.6)
Unknown	3 (2.1)
Sufficient anticoagulation	
Adjudicated sufficient	62 (44.3)
Adjudicated insufficient	76 (54.3)
Unknown	2 (1.4)
First timepoint of sufficient anticoagulation	
>48 h before eCV	10 (7.1)
24–48 h before eCV	9 (6.4)
12–24 h before eCV	5 (3.6)
1–12 h before eCV	24 (17.1)
<1h before eCV	14 (10.0)
After eCV/never	77 (55.0)
Unknown	1 (0.7)
INR at the time of eCV	
<1.5	88 (62.9)
1.5–2.0	25 (17.9)
>2.0	23 (16.4)
Unknown	4 (2.9)
PTT at the time of eCV	
<40 sec	68 (48.9)
40–60 sec	39 (27.9)
>60 sec	29 (20.7)
Unknown	4 (2.9)

Values are depicted as absolute numbers (percentage of total numbers).

eCV = electrical cardioversion

INR = international normalized ratio.

PTT = partial thromboplastin time.

TEE = transesophageal echocardiography.

The primary endpoint of new ischemic stroke or systemic embolism after cardioversion was met in 3 patients (2.1 %, 1 patient with ischemic stroke, 1 patient with systemic embolism, 1 patient with ischemic stroke and systemic embolism, Fig. 1). All 3 patients had new onset AF and insufficient anticoagulation at the time of cardioversion until diagnosis of stroke was made 3–17 days after cardioversion. Two of the three patients with new ischemic events had LAA thrombi excluded by TEE prior to cardioversion: One TEE study was not available for review. Following unsuccessful cardioversion for atrial fibrillation, the patient developed atrial flutter and underwent ablation of the cavotricuspid isthmus. The diagnosis of embolic stroke was made one week thereafter by computed tomography, which was initiated due to delayed wake-up response. The second patient’s TEE study was available for review and revealed no LAA thrombus or sludge. After TEE, the patient had several days of insufficient anticoagulation and developed retinal ischemia almost two weeks after cardioversion.

Two patients experienced an ischemic stroke prior to cardioversion. Four patients developed femoral artery occlusion in association with the decannulation and access site closure of a veno-arterial extracorporeal membrane oxygenation system. The events were hence not considered systemic embolism. One patient developed mesenteric ischemia without evidence of cardioembolism, which was considered to be caused by hypoperfusion.

**Fig. 2:**
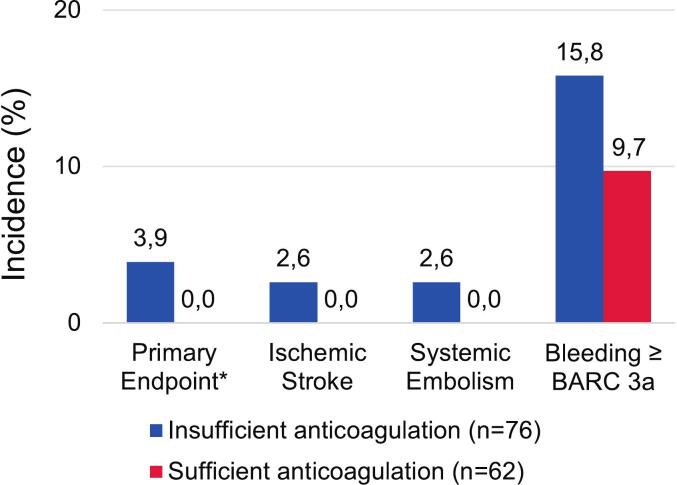
**Endpoints according to anticoagulation at the time of cardioversion.** Incidences of the primary endpoint, its components as well as Bleeding ≥ Type 3a according to BARC3 criteria.*one patient experienced both thromboembolic stroke and systemic embolism.

Endpoints stratified for sufficiency of anticoagulation are shown in Fig. 2. The all-cause mortality rate at 30 days was 37.9 % and the median length of ICU stay was 11.5 (5.0–18.0) days. Bleeding ≥ type 3a according to BARC criteria was found in 18 (12.9 %) patients after cardioversion. When restricted to patients discharged alive (n = 90), the primary endpoint occurred in 3 patients (6.0 %) without insufficient anticoagulation vs. none with sufficient anticoagulation, whereas bleeding ≥ type 3a occurred in 7 patients (14.0 %) without sufficient vs. 2 patients (5.3 %) with sufficient anticoagulation.

To enable analysis of potential risk factors for thromboembolic events in CS patients, we performed a regression analysis including all 16 patients with diagnosis of LAA-thrombus that did not undergo electrical cardioversion (Supplementary Fig. 1). History of atrial fibrillation and chronic heart failure were identified as predictors of thromboembolic events or LAA thrombus formation.

## 4 Discussion

This is the first study investigating thromboembolic events in CS patients undergoing cardioversion for AF or flutter. As main findings, the primary composite endpoint of ischemic stroke or systemic embolism was rare despite a high thromboembolic risk. All patients with ischemic stroke or systemic embolism had insufficient anticoagulation at the time of cardioversion. Yet, two of three patients meeting the primary endpoint did so regardless of negative TEE studies prior to cardioversion.

Patients in this study were heterogeneous and comprised patients with CS in the context of acute and non-acute coronary syndromes, valvular heart disease, and arrhythmias. All patients were critically ill with a high percentage requiring mechanical ventilation and mechanical circulatory support. A majority of patients had a CHA_2_DS_2_-VASc score ≥ 4 points suggesting a high thromboembolic risk despite the limited performance of the score in critically ill patients [11,12]. Contrasting the severity of disease, the observed incidence of ischemic stroke and systemic embolism was unexpectedly low. In a sub-study (n = 65) of the prospective AFTER-ICU study, 4.6 % of general ICU patients with new onset AF undergoing electrical cardioversion developed ischemic stroke, which might be explained by their low rate of anticoagulation (43 %) [13]. These findings did not differ significantly from the unstratified AFTER-ICU cohort (n = 423) including patients without electrical cardioversion, where the incidence of stroke was 4.5 % and the anticoagulation rate was 41 % [14]. Two sub-studies of randomized trials investigating patients with CS complicating myocardial infarction showed no difference in stroke rates between patients with or without AF [3,4]. In these two trials, stroke rates among patients with AF ranged from 1-7 % and the CHA_2_DS_2_-VASc score seemed comparable to our study, but cardioversion data was not reported. Another study of atrial fibrillation in critical care patients found no excess of thromboembolic events, but electrical cardioversion was performed in only 9.5 % [15].

There are several potential explanations for the low incidence of ischemic events in our study. Firstly, 74 % of the patients had new onset AF during the ICU stay. These patients hence likely had a short duration of AF at the time of cardioversion, a fact which is associated with a lower risk of developing left atrial thrombi [2]. Secondly, patients on mechanical circulatory support commonly receive high doses of anticoagulation to prevent extracorporeal clotting which in turn should also reduce the risk of developing atrial thrombi. Thirdly, the high mortality and high rates of ventilation of critically ill CS patients could lead to a low rate of reported ischemic events as some strokes events might have remained clinically inapparent. However, patients with CS are at high risk of stroke for other reasons such as left ventricular thrombi and mechanical circulatory support that may lead to overestimation of stroke rates.

Anticoagulation is an established means of stroke prevention in outpatients with AF, and particularly important within the first weeks after cardioversion. Its benefit in ICU patients is less clear and several studies raise concerns about an increased bleeding risk [11,[15], [16], [17], [18]]. Most patients in our study received unfractionated heparin for anticoagulation, necessitating constant vigilance of the partial thromboplastin time for adequate dosing. Many of these had insufficient anticoagulation at the time of cardioversion, including all patients with ischemic events, or received anticoagulation doses considered sufficient only shortly prior to cardioversion. An explanation for the low rate of sufficient anticoagulation may be the high number of acute onset atrial arrhythmias with immediate cardioversion or contraindications against full dose anticoagulation such as bleeding. Compared to published data, however, rates of anticoagulation in our study group were high. A systematic review of general ICU patients reported anticoagulation rates of only 17–50 %, possibly due to a higher anticipated bleeding risk [16]. Other studies found no benefit of anticoagulation on thromboembolic events in critically ill patients despite the cost of increased bleeding rates [11,[15], [16], [17], [18]]. Whether this increased bleeding risk in general and surgical ICU patients can be extrapolated to CS patients on a cardiac ICU is questionable. In our cohort, bleeding rates were remarkably high, but vigorous volume resuscitation in CS patients with subsequently observed hemoglobin drop might lead to an overestimation of bleeding rates in a retrospective analysis. Yet, the absolute number of events in this study was too low to perform a meaningful statistical analysis. Nevertheless, the numerically higher bleeding rate among patients with insufficient anticoagulation underscores that fluctuating or inadequate PTT values represent an ongoing concern. Vigilance in maintaining therapeutic anticoagulation levels is warranted, as insufficient or inconsistent anticoagulation may signal increased risk not only for stroke but also for bleeding. Considering the high prevalence of AF in ICU patients, further data from larger, prospective studies is warranted to establish guidance on anticoagulation management in these patients.

Whereas guidelines recommend TEE to exclude LAA thrombi to accept a shorter duration of anticoagulation prior to cardioversion in outpatients, its use in ICU patients has not been studied. In our study, two of the three patients experiencing ischemic events had no LAA thrombi in the TEE study prior to cardioversion. In turn, 16 patients in our registry had LAA thrombi revealed by TEE, which potentially prevented a cardioversion-induced stroke. Regression analysis identified chronic heart failure as significant risk factor for thromboembolic events. In contrast, no association was found between thromboembolic risk and acute illness severity, as measured by lactate levels, the Society for Cardiovascular Angiography & Interventions (SCAI) stage or the need for mechanical circulatory support. Therefore, caution should be warranted during emergency cardioversions for patients with a chronic disease course of heart failure or atrial fibrillation, whereas shorter disease courses may be associated with a lower risk of thromboembolic events.

In summary, given the considerable rate of LAA thrombus detection in our cohort, TEE prior to electrical cardioversion appears to be warranted to reduce thromboembolic risk. However, potential risks and benefits should be carefully weighed, particularly considering the duration of heart failure and atrial fibrillation, the adequacy of anticoagulation and whether the delay caused by performing TEE is appropriate for the patient’s hemodynamic condition.

## 5 Limitations

The main limitation of this study is the retrospective, single center design and the resulting sample size. Planned cardioversions that were aborted due to evidence of thrombus in TEE were not included in the analysis and information on the further course of these patients was unavailable. High rates of mortality and ventilation might have masked stroke events. Lastly, our study did not compare the effectiveness of rate control as opposed to rhythm control strategies in CS patients with AF.

## 6 Conclusion

In conclusion, electrical cardioversion of CS patients on the ICU was associated with a low incidence of thromboembolic events despite a high predicted thromboembolic risk, high rates of insufficient anticoagulation at the time of cardioversion, and low rates of TEE to rule out left atrial thrombi. Further research is warranted to establish anticoagulation strategies tailored to this patient group.

## CRediT authorship contribution statement

**Jonas Gmeiner:** Writing – review & editing, Writing – original draft, Formal analysis, Data curation, Conceptualization. **Lea Haag:** Investigation. **Enzo Lüsebrink:** Investigation. **Jan-Philipp Simon:** Investigation. **Felix Michelson:** Investigation. **Elina Oesterhaus:** Investigation. **Wolf-Stephan Rudi:** Writing – review & editing, Investigation. **Ludwig Weckbach:** Investigation. **Stefan Kääb:** Writing – review & editing, Supervision. **Michael Näbauer:** Writing – review & editing, Supervision. **Sven Peterß:** Writing – review & editing, Supervision. **Christopher Stremmel:** Writing – review & editing, Supervision. **Steffen Massberg:** Writing – review & editing, Supervision. **Martin Orban:** Writing – review & editing, Supervision. **Moritz F. Sinner:** Writing – review & editing, Supervision. **Clemens Scherer:** Writing – review & editing, Validation, Software, Resources, Project administration, Methodology, Funding acquisition, Formal analysis, Data curation, Conceptualization.

## Informed consent

Informed consent was obtained from all patients according to local ethics committee requirements.

## Funding

This work was supported by the Deutsche Forschungsgemeinschaft (DFG, 413635475) and Munich Clinician Scientist Program (MCSP) of LMU Munich (Clemens Scherer). The work was further supported by the Medical Faculty of the LMU Munich (Lea Haag, Elina Oesterhaus, Jan-Philipp Simon, Felix Michelson).

## Declaration of competing interest

The authors declare the following financial interests/personal relationships which may be considered as potential competing interests: Martin Orban: Speaker honoraria from Abbott Medical, AstraZeneca, Abiomed, Bayer vital, BIOTRONIK, Bristol-Myers Squibb, CytoSorbents, Daiichi Sankyo Deutschland, Edwards Lifesciences Services, Sedana Medical, support for attending meetings from AstraZeneca, Stocks from Abbott Laboratories, Abbvie, AstraZeneca, Bayer, Biontech, Bristol-Meyer- Squibb, Curevac, Draegerwerk, Fresenius Medical Care, Gilead sciences, Inari Medical, Johnson&Johnson, Linde, Merck US, Moderna, NovoNordisk, Nuance Communications, Pfizer, Proctor&Gamble, Roche, SAP, Siemens healthiniers, Zoom. Clemens Scherer: Speaker honoraria from AstraZeneca. Ludwig Weckbach: Speaker honoraria from AstraZeneca and Bayer. Sven Peterß: Speaker honoraria and travel compensation from Edwards Lifesciences Services, AstraZeneca, CytoSorbents, Terumo Aortic and CryoLife (Artivion) Remaining authors: no conflicts of interest to declare.
