# Supplementary material for: Thromboembolic risk of electrical cardioversion in patients with cardiogenic shock
Source: Int J Cardiol Heart Vasc. 2025 Nov 6;61:101835. doi: 10.1016/j.ijcha.2025.101835 (PMC12744523; doi:10.1016/j.ijcha.2025.101835)
Supplement: Supplementary Data 1 [file mmc1.docx]

Supplementary Table 1: Multivariable regression analysis of the extended cohort including patients diagnosed with LAA thrombus.

|  | Univariable | | Multivariable | |
| --- | --- | --- | --- | --- |
| Risk factor | Odds Ratio | p-value | Odds Ratio | p-value |
| Age | 0.998 (0.961-1.036) | 0.901 |  |  |
| Female Sex | 0.912 (0.282-2.948) | 0.877 |  |  |
| H/o Stroke | 1.225 (0.252-5.952) | 0.801 |  |  |
| CAD | 0.856 (0.306-2.395) | 0.767 |  |  |
| Chronic Heart Failure | 3.079 (1.138-8.331) | **0.027** | 2.591 (0.924-7.263) | **0.030** |
| Diabetes | 0.584 (0.183-1.876) | 0.364 |  |  |
| SAPS2 Score | 0.993 (0.948-1.040) | 0.755 |  |  |
| CHADS-VASC | 0.829 (0.606-1.135) | 0.243 |  |  |
| Highes Lactate Admission | 0.943 (0.849-1.047) | 0.270 |  |  |
| H/o Afib | 3.800 (1.058-13.654) | **0.041** | 3.221 (0.869-11.936) | 0.080 |
| SCAI stage | 1.997 (0.379-10.522) | 0.415 |  |  |
| Mechanical circulatory support (va-ECMO, Impella) | 1.002 (0.337-2.979) | 0.997 |  |  |

Supplementary Table 1 shows a regression analysis of all patients undergoing electrical cardioversion as well as patients with diagnosis of left atrial appendage (LAA) thrombus not undergoing electrical cardioversion (n=156) to explore potential risk factors for thromboembolic risk.
